# Cancer Therapeutic Targeting of Hypoxia Induced Carbonic Anhydrase IX: From Bench to Bedside

**DOI:** 10.3390/cancers14143297

**Published:** 2022-07-06

**Authors:** Paul C. McDonald, Shawn C. Chafe, Claudiu T. Supuran, Shoukat Dedhar

**Affiliations:** 1 Department of Integrative Oncology, BC Cancer Research Institute, Vancouver, BC V5Z 1L3, Canada; pmcdonal@bccrc.ca; 2 Centre for Discovery in Cancer Research, McMaster University, Hamilton, ON L8S 4K1, Canada; chafes@mcmaster.ca; 3 Department of Neurofarba, Section of Pharmaceutical and Nutraceutical Sciences, University of Florence, Polo Scientifico, Via U. Schiff 6, Sesto Fiorentino, 50019 Firenze, Italy; claudiu.supuran@unifi.it; 4 Department of Biochemistry and Molecular Biology, University of British Columbia, Vancouver, BC V6T 1Z3, Canada

**Keywords:** hypoxia, Carbonic Anhydrase IX, immunotherapy, small molecule inhibitor, SLC-0111, acidosis, combination therapy, ferroptosis

## Abstract

**Simple Summary:**

Tumor hypoxia remains a significant problem in the effective treatment of most cancers. Tumor cells within hypoxic niches tend to be largely resistant to most therapeutic modalities, and adaptation of the cells within the hypoxic microenvironment imparts the cells with aggressive, invasive behavior. Thus, a major goal of successful cancer therapy should be the eradication of hypoxic tumor cells. Carbonic Anhydrase IX (CAIX) is an exquisitely hypoxia induced protein, selectively expressed on hypoxic tumor cells, and thus has garnered significant attention as a therapeutic target. In this Commentary, we discuss the current status of targeting CAIX, and future strategies for effective, durable cancer treatment.

**Abstract:**

Carbonic Anhydrase IX (CAIX) is a major metabolic effector of tumor hypoxia and regulates intra- and extracellular pH and acidosis. Significant advances have been made recently in the development of therapeutic targeting of CAIX. These approaches include antibody-based immunotherapy, as well as use of antibodies to deliver toxic and radioactive payloads. In addition, a large number of small molecule inhibitors which inhibit the enzymatic activity of CAIX have been described. In this commentary, we highlight the current status of strategies targeting CAIX in both the pre-clinical and clinical space, and discuss future perspectives that leverage inhibition of CAIX in combination with additional targeted therapies to enable effective, durable approaches for cancer therapy.

## 1. Introduction

Carbonic Anhydrase IX (CAIX), a strong effector of hypoxia, is expressed on the cell surface of tumor cells that reside within hypoxic niches. Because this enzyme is rarely expressed on normal cells within the body, it has received considerable attention as a cancer-specific therapeutic target.

The extrafacial catalytic domain of CAIX can reversibly convert carbon dioxide to bicarbonate and protons, the former internalized by sodium-bicarbonate transporters to buffer the acidic intracellular pH of hypoxic tumor cells, while the protons contribute to the acidic extracellular microenvironment and promote tumor cell invasion [1,2,3,4,5].

## 2. Immunotherapy

Significant research activity to target CAIX with both antibodies and small molecule inhibitors has progressed to the clinic [6]. A CAIX specific monoclonal antibody, Girentuximab (trade name Rencarex), has been evaluated in Phase III clinical trials [7], but while the trial results did not meet the criteria for clinical approval, it was clear that some patients would see benefit from targeting CAIX. These findings have spurred interest in using anti-CAIX antibodies to deliver radioligands and toxic payloads as antibody-drug conjugates (ADC), including evaluation of a monomethyl auristatin E-based ADC, BAY 79-4620, in a Phase I clinical trial [8,9,10]. Similar payload-based strategies are being developed using small molecules targeting CAIX for delivery of therapeutic agents [11,12]. Immunotherapy approaches using CAIX-targeted chimeric antigen receptor T (CAR-T) cells are also currently gaining traction, with the development of new generations of CAIX CAR-T cells resulting in the initiation of Phase I clinical trials evaluating these agents for treatment of advanced renal cell carcinoma (NCT04969354) [13,14,15]. Administration to renal cancer patients of autologous dendritic cells transduced by adenovirus with a granulocyte-macrophage colony-stimulating factor/CAIX fusion construct (DC-AdGMCAIX) elicited a CAIX-specific immune response in Phase I trials, further highlighting the potential promise of CAIX-targeted immune therapy [16]. The recent development of several novel monoclonal antibodies targeting CAIX [9,17,18], with differential properties to inhibit catalytic activity, superior internalization properties, and favorable properties for positron emission tomography (PET) imaging, bode well for the future development of clinical targeting of CAIX using anti-CAIX antibodies.

## 3. Small Molecule Inhibitors

The development of small molecule inhibitors of CAIX has also been intensely pursued in recent years, aided by an increased understanding of the biology of CAIX, which has revealed that hypoxia induced CAIX is strongly expressed on cancer stem cells (CSC), and inhibiting CAIX leads to depletion of such cells, resulting in suppression of tumor progression and metastasis [19,20,21,22]. Additionally, CAIX has been identified as a protein that can associate with integrins and the membrane bound matrix metalloproteinase-14 (MMP-14), and regulate cell adhesion, migration and invasion [23]. CAIX is intimately connected to the actin cytoskeleton [24,25] and with components of invadopodia of aggressive tumor cells [23,26,27]. Recent work has also demonstrated a central role of CAIX in regulating tumor metabolism, by influencing lactate transport through the monocarboxylate transporters (MCT1 and 4) [28,29,30,31], as well as regulation of iron–sulfur clustering and components of the ferroptosis cell death pathway [32].

These biological insights have significantly influenced the evaluation of small molecule inhibitors of CAIX. While there are now a large number of inhibitors of CAIX activity (reviewed recently in [33]), the most successful class of compounds in terms of clinical development have been the uriedo-substituted benzene sulfonamides, first identified in 2011 as highly selective and potent inhibitors of CAIX [34,35]. A lead compound from this class of inhibitors, SLC-0111, has been tested in a Phase I clinical trial in patients with advanced disease [36]. In this trial, the compound was found to have acceptable safety and pharmacokinetic profiles, and a maximum tolerated dose (MTD) was identified for Phase II trials. SLC-0111 is also currently being evaluated in a Phase Ib clinical trial in pancreatic cancer patients with CAIX-positive tumors in combination with gemcitabine (NCT03450018).

The benzene sulfonamide scaffold may be particularly relevant for the inhibition of CAIX, as this scaffold was identified as recently in a large DNA Expressed Library (DEL) screen combined with artificial intelligence (AI) machine learning strategies to identify CAIX inhibitors [37]. Further screening using such approaches may identify additional “druggable” compounds for specific inhibition of CAIX.

A critical element to be considered for the development of therapeutic inhibitors of CAIX is that, because of the restricted expression of CAIX on cancer cells within the hypoxic niche, it is unlikely that CAIX inhibitors will have significant effects on tumor growth and progression as monotherapy agents. This has been borne out in a number of in vitro and in vivo tumor models. Combining CAIX inhibitors with standard of care chemotherapy agents has been demonstrated to be significantly more efficacious than single agents alone [38,39,40]. Thus, combining the CAIX inhibitor, SLC-0111 with paclitaxel or with sunitinib in breast cancer [19,41], with temozolomide in pediatric and adult glioblastoma [42], with gemicitabine in pancreatic cancer [43], or an analog of SLC-0111, FC531, with cytarabine or Quizartinib in acute myeloblastic leukemia (AML) [44], results in greater inhibition of tumor growth and in significant increases in the survival of the tumor bearing mice.

These pre-clinical models demonstrate that CAIX inhibition may sensitize tumor cells to cytotoxic agents, although the mechanisms of such sensitization are not clear. It is also possible that increased efficacy of the combinations is apparent because the CAIX inhibitors target the residual cancer cells within hypoxic niche which are resistant to the cytotoxic agents, resulting in more durable suppression of tumor growth. The combinations also result in suppression of metastasis in many cases, due to the specific effects of inhibiting CAIX on CSCs and on inhibiting tumor cell migration and invasion [19,42].

While the above-described studies show improved response of these combinatorial approaches, resistance mechanisms result in tumor recurrence and, ultimately, systemic growth and morbidity [43].

**Figure 1:**
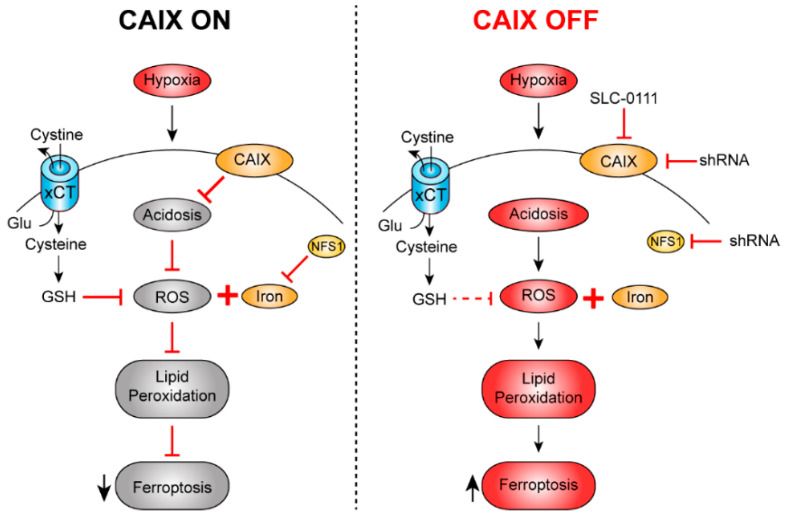
Hypoxia-induced CAIX activity and iron–sulfur clustering are co-vulnerabilities that can be targeted in combination to induce ferroptosis-mediated death of cancer cells. When CAIX is active (CAIX ON) in the presence of NFS1, intracellular pH and reactive oxygen species (ROS) are effectively regulated, resulting in low levels of lipid peroxidation and inhibition of ferroptosis. Combinatorial inhibition of CAIX activity (CAIX OFF) and NFS1 expression results in intracellular acidosis and deregulation of iron-sulfur clustering which leads to increased ROS, induction of lipid peroxidation and promotion of ferroptosis. Treatment strategies designed to inhibit CAIX and induce ferroptosis have the potential to overcome therapeutic resistance in hypoxic tumors. GSH, glutathione; Glu, glutamate.

To identify potential co-vulnerabilities of hypoxic tumor cells to inhibition of CAIX, which could be exploited to prevent, or suppress, resistance and recurrence, a recent genome-wide synthetic lethal approach in triple negative breast cancer cells revealed that hypoxia induced CAIX activity suppresses ferroptosis mediated cell death, and that inhibiting CAIX renders tumor cells more vulnerable to compounds that induce ferroptosis [32]. Specifically, components of iron–sulfur clustering, which regulate ferroptosis, such as cystine/glutamate antiporter xCT and NFS1 cysteine desulfurase (NFS1), were identified as co-vulnerabilities of CAIX inhibition, identifying novel combinatorial therapeutic strategies with CAIX inhibition and ferroptosis inducers. These findings provide novel, exciting combinatorial strategies to effectively induce cell death in hypoxic tumors (Figure 1).

The enhanced efficacy of the combinations with CAIX inhibitors is not restricted to small molecule cytotoxic compounds, but also to immune checkpoint inhibitors. For example, CAIX inhibition with SLC-0111 significantly enhanced the efficacy of anti-PD1/anti-CTLA4 immunotherapy in preclinical models of melanoma and triple negative breast cancer [45]. Inhibiting CAIX results in the neutralization of the extracellular acidic pH, thus improving T-cell function and reducing the T-Reg cells within the tumor microenvironment [45]. More studies are warranted in additional immunotherapy models to grasp the potential of CAIX inhibitors in improving immune checkpoint inhibitor effectiveness.

Acidosis of the tumor microenvironment has been clearly demonstrated to be immunosuppressive [46,47,48], and attempts to reverse acidosis in pre-clinical models using systemic administration of bicarbonate [48,49,50] has demonstrated that buffering extracellular acidosis can reduce immunosuppression. Efforts to suppress acidosis by directly reducing acidosis, or by inhibiting lactic acid extrusion by inhibiting the moncarboxylate transporters, MCT1 and 4, or suppressing proton accumulation by inhibiting CAIX, are strategies that need to be developed in the future to improve cancer immunotherapy approaches.

Hypoxia also results in HIF-1 dependent cell surface expression of the nucleotidase, cluster of differentiation 73 (CD73), which converts adenosine monophosphate (AMP) to adenosine, a potent immunosuppressor [51]. Anti-CD73 monoclonal antibodies such as CPI-006, and small molecule inhibitors of CD73 such as etrumadenant (AB928), show promise in pre-clinical models and early phase clinical trials [51]. It will be interesting to determine whether combinations of these inhibitors with inhibition of CAIX or MCTs will have stronger anti-tumor effects. A further consideration is the development of novel small molecules capable of combined CAIX and CD73 inhibitory activity, given that both enzymes are zinc coordinated and some CAIX inhibitors have been shown to inhibit CD73 [52,53].

## 4. Conclusions

In conclusion, the last ten years have witnessed remarkable progress in targeting the CAIX/pH regulation axis, with the development of antibody and small molecule therapeutic strategies. As discussed, these novel agents are unlikely to be useful as single agents, but in combination with chemotherapy and immunotherapy, they could achieve significant improvements in the treatment of difficult to treat hypoxic tumors.
